# Increased male bias in eider ducks can be explained by sex-specific survival of prime-age breeders

**DOI:** 10.1371/journal.pone.0195415

**Published:** 2018-04-10

**Authors:** Satu Ramula, Markus Öst, Andreas Lindén, Patrik Karell, Mikael Kilpi

**Affiliations:** 1 Department of Biology, University of Turku, Turku, Finland; 2 Bioeconomy Research Team, Novia University of Applied Sciences, Ekenäs, Finland; 3 Environmental and Marine Biology, Faculty of Science and Engineering, Åbo Akademi University, Turku, Finland; Université de Sherbrooke, CANADA

## Abstract

In contrast to theoretical predictions of even adult sex ratios, males are dominating in many bird populations. Such bias among adults may be critical to population growth and viability. Nevertheless, demographic mechanisms for biased adult sex ratios are still poorly understood. Here, we examined potential demographic mechanisms for the recent dramatic shift from a slight female bias among adult eider ducks (*Somateria mollissima*) to a male bias (about 65% males) in the Baltic Sea, where the species is currently declining. We analysed a nine-year dataset on offspring sex ratio at hatching based on molecularly sexed ducklings of individually known mothers. Moreover, using demographic data from long-term individual-based capture-recapture records, we investigated how sex-specific survival at different ages after fledgling can modify the adult sex ratio. More specifically, we constructed a stochastic two-sex matrix population model and simulated scenarios of different survival probabilities for males and females. We found that sex ratio at hatching was slightly female-biased (52.8%) and therefore unlikely to explain the observed male bias among adult birds. Our stochastic simulations with higher survival for males than for females revealed that despite a slight female bias at hatching, study populations shifted to a male-biased adult sex ratio (> 60% males) in a few decades. This shift was driven by prime reproductive-age individuals (≥5-year-old), with sex-specific survival of younger age classes playing a minor role. Hence, different age classes contributed disproportionally to population dynamics. We argue that an alternative explanation for the observed male dominance among adults–sex-biased dispersal–can be considered redundant and is unlikely, given the ecology of the species. The present study highlights the importance of considering population structure and age-specific vital rates when assessing population dynamics and management targets.

## Introduction

Adult sex ratio (ASR; the proportion of adult males in the adult population) is a key demographic parameter influencing population growth, extinction, and individual behaviour [1–4]. Despite this importance, progress in understanding ASR dynamics was long hampered by the previously held paradigm that ASR fluctuations are tightly regulated [5] because intensifying intrasexual competition in the more common sex was thought to buffer against any deviation from an even sex ratio (e.g., [6]). More recently, however, there has been a growing realization that ASR bias is ubiquitous in nature (reviewed in [7]).

Male-biased ASR is the norm in birds [8] and this skew is particularly pronounced in endangered species with small population sizes [9]. Nonetheless, empirical studies exploring spatial and temporal variation in ASR within species are rare (but see [10,11]). Due to this paucity of research, there is no consensus about the relative contributions of different demographic mechanisms to observed biases in ASR [12]. The majority of avian studies suggest that ASR is a product of differential selection on adult males and females owing to sexual differences in breeding behaviour and life histories [9,13] as sex ratios at hatching are often even (reviewed in [9]). For example, threatened species (according to IUCN criteria) for which introduced predators are listed as a severe threat have significantly more male-biased ASRs than other threatened species [9], presumably because incubating females may be disproportionally affected by predation. However, some recent studies have challenged this notion by demonstrating that male-biased ASRs in birds may primarily reflect sex differences in juvenile rather than in adult mortality [12,14]. Yet, another explanation for shifts in ASR can be sex-biased dispersal [3]. Clearly then, there is a need for greater clarity about the point in life history at which ASR biases emerge, as well as their mechanisms.

Heeding this call, we examined the origin of the recently documented dramatic temporal change in the ASR of eider ducks (*Somateria mollissima*) breeding in the Baltic Sea [15]. In monogamous eiders, the proportion of adult males in the Baltic Sea has increased during the past three decades and, consequently, the ASR has reversed from a slight female bias to a strong male bias (currently about 65% males; Öst et al., unpublished). This shift may be due to changes in the sex ratio already at birth (but see [16]), sex differences in the survival of juveniles [16], or reduced survival of breeding females due to increased stress and/or predation, particularly by the white-tailed sea eagle (*Haliaeetus albicilla*), an apex predator rapidly rebounding from a pesticide-related decline [15,17,18]. Previously, Lehikoinen et al. [15] stated that an 11% difference in mortality between the sexes would explain the observed change in the ASR of eiders. However, this estimate did not consider population age structure or the fact that individuals across different ages contribute disproportionally to population dynamics. For example, in their study on the decline of female eiders in the Baltic Sea area, Öst et al. [19] showed that the population growth rate of females is far more sensitive to small proportional changes in the mortality of older, experienced breeders than to those of younger age classes. Such a pattern is typical for long-lived vertebrates, including many birds [12, 20] and is due to the greater reproductive value of older, reproductive individuals that have high survival [21]. Therefore, population models that explicitly consider age structure are necessary for exploring the demographic mechanisms of changes in ASR. Due to the lack of data on sex-biased dispersal for the eider, we do not analyze its potential effect in this study, but discuss its plausibility as an explanation for the observed change in ASR.

Here, we addressed the following questions: (1) Can the observed change in ASR be due to shifts toward a male bias at hatching? (2) Can sex-specific survival later in life explain the male-biased sex ratio? (3) If so, in which age class(es) is the survival difference between the sexes most critical for explaining the observed shift in ASR? To this end, we first analysed a nine-year data set (2005–2013) on offspring sex ratios at hatching from Tvärminne, a well-studied eider population in the western Gulf of Finland, Baltic Sea. We then used an age-structured population model (a stochastic two-sex matrix model) to investigate potential mechanisms for the observed male-biased ASR of eiders. We predicted that offspring sex ratio at hatching would be independent of the prevailing ASR. This is because theory suggests that parental compensation is not expected if skewed sex ratios emerge after the end of the parental investment period [6,22]. We also predicted that sex-specific survival among prime-age breeders would make the largest relative contribution to the observed ASR bias because the long-term population growth rate of female eiders is most sensitive to small relative perturbations in the survival of experienced breeders in the Baltic Sea population [19].

## Methods

### Study sites

In our analyses, we used published estimates of demographic parameters (Öst et al. 2016), based on long-term individual-based capture-recapture data from Söderskär (60°07’N, 25°25’N), central Gulf of Finland, collected during 1986–2003, and from Tvärminne (59°50’N, 23°15’E), western Gulf of Finland, collected during 1997–2010. The distance between the sites is about 130 km. The Tvärminne site represents the core breeding area of the species in Finland, and was further divided into forested and open (rocky) habitat types (see [18] for a detailed site description), hereafter termed Tvärminne_forested_ and Tvärminne_open_, respectively. These two population fractions differ in survival, with adult female survival being significantly lower in the open than in forested habitats ([18,19]; see Table 1 for parameters).

**Table 1:** Vital rates used to simulate change in the adult sex ratio of eiders in Söderskär and Tvärminne populations that differ in fecundity and female survival.

Estimates	Söderskär	Tvärminne_forested_	Tvärminne_open_
*F*	0.08 ± 0.025	0.75 ± 0.129	0.75 ± 0.126
Annual SD of *F*	0.098	0.452	0.452
*S*_*f*2–5_	0.86 ± 0.011	0.76 ± 0.039	0.60 ± 0.044
Annual SD of *S*_f2–5_	0.007	0.094	0.032
*P*_2_	0.09	0.09	0.09
*P*_3_	0.36	0.36	0.36
*P*_4_	0.38	0.38	0.38
*P*_5_	0.17	0.17	0.17
*R*	0.47 ± 0.013	0.47 ± 0.013	0.47 ± 0.013
Annual SD of *R*	0.065	0.065	0.065
*S*_*j*_	0.50	0.50	0.50
*S*_*f*1_	0.70	0.70	0.70
*S*_*m1-5*_	0.88, 0.90 or 0.92	0.88, 0.90 or 0.92	0.88, 0.90 or 0.92
ln λ_s_	–0.120…–0.090	–0.097…–0.075	–0.135…–0.093

Means ± SEs for fecundity (*F*) and survival of different age classes for females (*S*_f2–5_), as well as their annual variation (SDs), while constant point estimates are applied to other vital rates. Abbreviations are: (*P*_2–5_) breeding probabilities for different age classes, (*R*) proportion of males among hatched ducklings, (*S*_*j*_) juvenile survival from hatchling to one-year of age, (*S*_*f*1_) female survival in age class 1, (*S*_m1–5_) male survival for different age classes. Ln λ_s_ denotes mean stochastic population growth rate calculated from simulations for 50 years with 100 runs assuming higher survival for males than for females simultaneously in age classes 1–5.

At both Söderskär and Tvärminne, the number of breeding females and the production of fledglings per adult female were recorded annually. The males were not caught, but their demographic parameters were obtained from the literature (see below). Incubating females were captured and ringed at the nest towards the end of the incubation period following the same capture protocol and methods at both sites; mean capture success was similar (> 50%) [19]. The annual average sample size of captured females was 655 at Söderskär [23] and 144 at Tvärminne [18]. Fledgling counts were conducted at a phenologically equivalent time at both sites. During these counts, the total number of ducklings, brood-tending adult females and solitary adult females was recorded, yielding estimates of annual fecundity per female for each population [19]. Eider populations at these two sites are predicted to have declined about 11–33% per year during the study period when assessed based on females only [19].

### Sex ratio at hatching

Ducklings of individually known mothers captured in the same season were sexed on the core study islands in Tvärminne during 2005–2013. As ducklings leave the nest within 24 hours of hatching, they were sampled during consecutive nest visits timed to coincide with the estimated hatching date based on egg floatation [24] performed at the time of female capture. A small blood sample (< 50 μl) was drawn from the tarsal vein of each duckling for further analyses. The total sample size consisted of 1916 successfully sexed ducklings (annual mean ± SD: 213 ± 116.1) from 519 different broods. The handling of females and their young was approved by the Animal Experiment Board/State Provincial Office of Southern Finland (permit numbers HY-85-2003, ESLH-2009-02969/Ym-23 and ESAVI/1697/04.10.03/2012), and complied with the regulations of Tvärminne Zoological Station. Duckling sex was then determined by molecular sexing which is based on the amplification of a part of the gene for the chromo-helicase DNA binding protein and yields different sized amplicons in female and male eiders [25]. Positive and negative controls were included for each PCR reaction, and samples with ambiguous results were rerun. The overall success rate of sexing was 91.8% (1916 out of 2087 ducklings).

To examine whether sex ratio at hatching differs from even and whether it has changed during the past decade, the duckling sex ratio, i.e. the probability of a duckling being male, was analysed statistically using a generalized linear mixed model (GLMM) with clutch size and year as fixed continuous explanatory variables (modelling the temporal trend). Both continuous variables were initially centralised to zero mean, by subtracting their sample averages. Two factor variables, female ID and year ID, were defined as random effects on the intercept to account for individual and annual correlation in the unexplained variation. To fit the model, we applied the function ‘glmer’ in the *lme4* package of R [26,27] with a maximum likelihood estimate based on a Laplace approximation. We used the optimizer BOBYQA and a maximum of 20000 function evaluations.

### Demographic model and stochastic simulations

**Fig 1:**
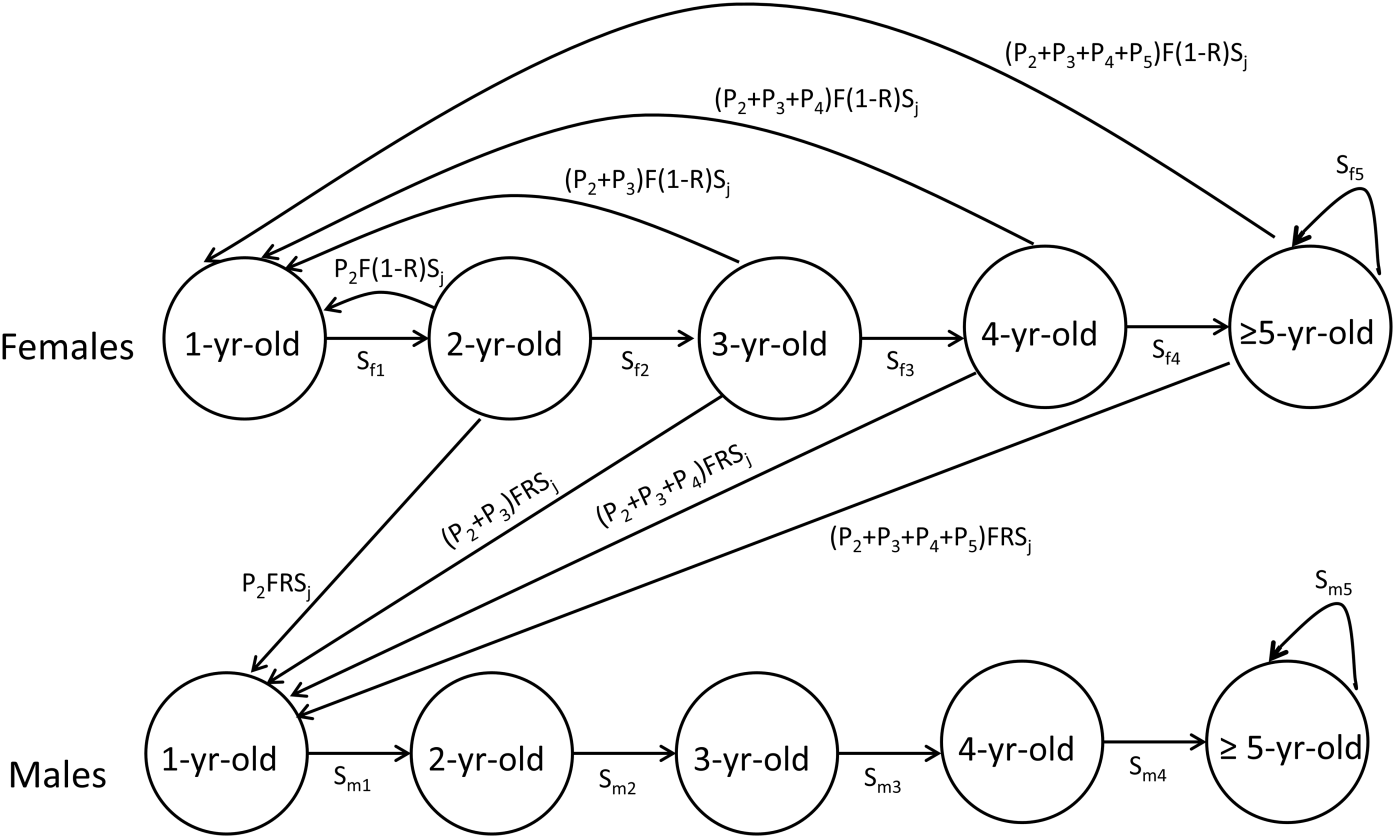
A two-sex age-structured population model for the eider. Vital rates are: breeding probabilities for different age classes (*P*_2–5_), proportion of males among hatched ducklings (*R*), juvenile survival from hatchling to one-year of age (*S*_*j*_), fecundity (*F*), survival of different age classes for females (*S*_f1–5_) and for males (*S*_m1–5_).

To explore how survival differences between the sexes after the duckling stage contribute to changes in ASR, we constructed an age-structured two-sex matrix population model. Two-sex matrix models consider the entire life-cycle of individuals from birth to death, and are suitable for predicting population dynamics over time when vital rates are sex-specific [21,28]. We adopted an age-structured model that was previously used to assess the long-term population growth rate of female eiders at the same study sites (Söderskär and Tvärminne, [19]). As this previous model was based on females only, i.e. a single-sex model [19], we expanded it to include males so that the model contained the following, equivalent five age classes for both sexes: 1-year-old individuals (non-reproductive), 2-, 3-, and 4-year-olds, and ≥ 5-year-old individuals (Fig 1). Breeding begins at the age of 2–5 years with a modal age-of-first-breeding of three years [23] and continues at least up to 17 years old without dramatic changes in clutch size with advancing age [29]; yearlings do not breed [23,29]. Although the oldest age class in the model contains individuals of different ages, most of them are still at their prime age (<10 years old) [30,31], with hatching success increasing with age [30]. In the model, fecundity was assumed to be independent of the relative proportions of the sexes in the population because there is currently no evidence that female reproductive success would be associated with ASR (M. Öst et al., unpubl.). Model parameters for juveniles and females are identical to those of Öst et al. [19] and the same logic is used for males. In brief, juvenile survival (*S*_j_) is constant, while females (*S*_*f2-5*_) and males (*S*_*m2-5*_) in older age classes survive at a certain probability, adult females breed with the probabilities of *P*_2_-*P*_5_ depending on their age class, and produce fledglings (*F*) of which 47% are males (*R*) and 53% are females (1 –*R*) (Fig 1, Table 1).

Due to differences in eider population dynamics at small spatial scales [18,19], we used demographic parameters from two closely located sites (Söderskär, Tvärminne), with Tvärminne divided further into two breeding habitat types (Tvärminne_forested_, Tvärminne_open_). In these populations, survival of adult females varied from 0.86 (Söderskär) to 0.60 (Tvärminne_open_) and fecundity was either extremely low (0.08 fledglings per female at Söderskär) or closer to one (0.75 at Tvärminne; Table 1). For Söderskär, we had no access to raw data and therefore, we estimated annual apparent survival of adult females directly from annual capture–recapture records on nesting females reported in [32], while for Tvärminne, we used a time-dependent Cormack–Jolly–Seber (CJS) model with a linear trend in recapture probability, applied to the capture-recapture data collected by our research team (see 19 for details). As no demographic data were available for males from the study sites, we adopted male survival estimates (mean = 0.92, CI = 0.87–0.95) from a Canadian population of eiders [33]. To consider uncertainty in male survival obtained from the literature, we used three different survival rates (88%, 90% or 92%) for males in age classes 1–5 in each population; the two lowest survival rates were chosen based on our long-term experience of the study populations. We note that somewhat higher or lower survival for males does not qualitatively affect our conclusions. Fecundity (*F*) was estimated from annual fledgling counts at each study site (see above). Breeding probabilities (*P*_2_–*P*_5_) were estimated from the Söderskär population using data from [23]. More specifically, mean breeding probabilities were calculated from Fig 1 in [23], which covered different population phases from a population increase to a population decline during 1975–2004. The same breeding probabilities were applied also to Tvärminne where age at first breeding is unknown. We expect breeding probabilities to be similar between the study sites because the decision to breed is not significantly associated with white-tailed sea eagle-induced predation risk (Öst et al., unpubl.), the type of predation primarily differentiating the two populations. Moreover, breeding probabilities remain constant regardless of population status (declining or increasing) [23]. Following Öst et al. [19], juvenile survival (*S*_*j*_) was set to be 0.5 in the models, but *S*_*j*_ varying between 0.1–0.7 had no major impact on the results. The survival of 1-year-old, non-breeding females (*S*_*f*1_) was set to be 0.70 in order to model survival as an increasing function of an individual’s age. This adjustment was done to increase model realism because in many bird species, including the closely related king eider (*S*. *spectabilis*), survival tends to be lower for hatch-year individuals than for older individuals (e.g., [34]). We note that the model outcomes are not sensitive to this female survival parameter within a range of 0.70–0.92 (results not shown). The proportion of males among hatched ducklings (*R*) was parameterized from the nine-year data from the Tvärminne site presented here (see Results), in which males were slightly underrepresented (47%, Table 1).

To explore change in population ASR over time, we simulated population dynamics for 50 years with 100 replicated runs by multiplying the transition matrix (**A**) of a given time step and population size (**n**), and calculated stochastic population growth rate (ln λ_s_) across the runs. To incorporate estimation uncertainty and annual variability of vital rates in the transition matrices, we simulated data as following. For each run, we first drew the average logit-scale survival and log-scale fecundity of females from a multinormal distribution based on their estimated means and covariance matrix (uncertainty of the averages). Moreover, we drew the average logit-scale sex ratio of hatched ducklings (males) from a normal distribution based on its estimated mean and variance. After this, we drew 50 annual parameter values, with the previously drawn mean and estimated annual covariance matrix, to incorporate temporal variation in vital rates during the simulation (see S1 Appendix for a computer code). Due to lack of data, the sex ratio of hatched ducklings was assumed to be non-correlated with survival and fecundity, and an annual estimate of this parameter was drawn independently of other vital rates based on the previously estimated mean. This simulation procedure thus included variation in these three vital rates among the replicated runs, as well as variation among years within each run. Fixed values (point estimates) were used for the remaining vital rates, including male survival with the three alternative levels (Table 1) simultaneously in age classes 1–5. To explore the contributions of different age classes to ASR, we then simulated higher male survival separately for each age class 1–5, assuming no survival difference between the sexes for other age classes (i.e. *S*_*m*_ was set to equal to *S*_*f*_). All simulations were initiated from the stable age distribution estimated from mean vital rates of a given simulation, with 47% males and 53% females in the youngest age class (1-year-old) and with an even sex ratio in older age classes. Changes in ASR over time were assessed based on the proportions of males and females in age classes 1–5 (S1 Appendix). Similar to the statistical analyses, the simulations were conducted in R 3.2.3 [27].

## Results

### Sex ratio at hatching

In the analysis of duckling sex ratio at hatching we found slight evidence for female bias with males being underrepresented (intercept: –0.112 ± 0.053 SE, Z = –2.1, *P* = 0.036). This corresponds to a proportion of 47.2% males (approximate 95% CI = {44.6%, 49.9%}). Neither an annual trend (b = 0.014 ± 0.021 SE, Z = 0.65, *P* = 0.516) nor clutch size (b = –0.043 ± 0.046 SE, Z = –0.93, *P* = 0.351) was able to significantly explain variation in duckling sex ratio. The random effect SD of female ID converged to zero, while that of annual variation was estimated to be 0.065, indicating negligible variation in offspring sex ratio among females within years.

### Sex-specific survival and adult sex ratio

**Fig 2:**
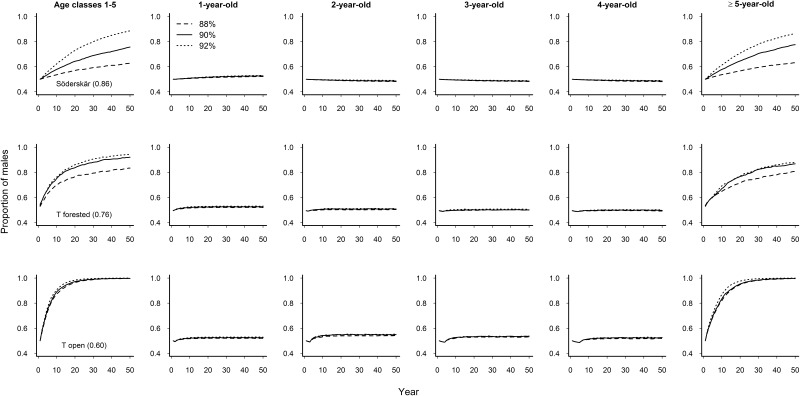
Mean adult male proportions of the eider resulting from stochastic simulations at three sites (in rows) with different survival probabilities for females (*S*_f2–5_ in parentheses) and for males (88%, 90% or 92%). Each scenario is based on 100 replicated runs for 50 years with three alternative male survival probabilities applied either simultaneously to age classes 1–5 (the first panel) or separately to each age class (panels 2–6). Error bars are omitted for clarity and T denotes the Tvärminne population.

When male survival was higher (2–32 percentage points) than female survival simultaneously in age classes 1–5, all populations were predicted to decline by 8%–14% annually (Table 1). Moreover, stochastic simulations revealed that under such conditions population ASR shifted from a slight female bias to a strong male bias (> 0.6 males) in about 5–20 years, depending on the initial survival difference between the sexes (Fig 2, the first panel). As predicted, age classes contributed disproportionally to the change in population ASR. During the simulation period of 50 years, change toward male dominance was clearly driven by sex-specific survival among prime reproductive-age individuals (≥5-year-old individuals; Fig 2). Survival differences between the sexes in younger age classes (1–4-year-old individuals) had a smaller impact on population ASR, with the proportion of males in the population being less than 0.55 in all populations (Fig 2, panels 2–5).

## Discussion

Our results were consistent with our prediction that the observed change from about an equal proportion of sexes to male bias in the adult population was primarily due to the reduced survival of prime reproductive-age female eiders (≥ 5-year-olds). While we have previously pointed out the crucial role of experienced breeders in the population growth rate of female eiders [19], the present study shows that the sex-specific survival of adult stages can be a key for understanding the ongoing change in population dynamics and ASR. This finding was further supported by the observation that sex ratio at hatching was slightly female-biased, indicating that most of the survival differences between the sexes indeed emerge later in life. Overall, our results are in line with the previous observation across wild bird species, for which adult survival is generally a better predictor of ASR than sex ratio at hatching [13]. However, the slightly female-biased sex ratio among hatched eider ducklings contrasts with our expectation that females do not vary their offspring sex ratio relative to ASR, for reasons discussed below.

### Sex ratio at hatching and sex differences in survival

While we found no evidence of sex ratio bias at hatching in our earlier analysis dating back to 2003 [16] when ASR was still less male-biased than at present, we detected a slight female bias (53%) among ducklings in the current analysis ending in 2013. Although the mechanism remains to be established, these observations are at least consistent with the hypothesis that female eiders may be capable of adjusting their offspring sex ratio relative to local ASR by producing more female ducklings when males are overrepresented at adult stages. This finding contrasts with the theoretical expectation that parental compensation should not occur if both sexes are equally costly to produce [6,22], which may be the case in eiders, in which the sexes do not differ regarding body mass or structural size at hatching [16]. Indeed, the bulk of empirical evidence suggests that even sex ratios at hatching are the norm in many birds (e.g., [35,36] but see e.g., [12]), including waterfowl [37]. However, our finding fits well with recent theoretical models that consider sex-specific survival at adult stages. For example, Shyu and Caswell [38] demonstrated that survival differences between the sexes after the period of parental investment have the potential to modify sex ratio at birth. Their theoretical simulations showed that the sex with lower adult survival is favoured at birth, while ASR will be biased toward the sex with higher adult survival. Nevertheless, it should be noted that overall, the long-term sex ratio at hatching in eiders from the Baltic Sea shows surprisingly little variation in light of the concurrent major and still ongoing shift in ASR.

Our simulations revealed that even a few percentage points (2 at the smallest) difference in adult survival between the sexes tends to result in a considerable change in population ASR within a few decades. As predicted, these simulations confirmed the disproportionate role of different age classes in the observed change in population ASR, with the survival of prime reproductive-age individuals (≥ 5-year-old) having a greater impact on ASR than that of younger age classes. It has been previously suggested that sex differences early in life rather than later in life are likely to contribute to biased ASRs in birds [12,14], but our simulations indicate that the opposite is true for eiders. The observed importance of adult survival can be explained by differences in reproductive value, which considers jointly survival and fecundity, between individuals of different ages. For long-lived animals, reproductive value tends to increase up to a certain age [21] and therefore, older, experienced breeders that exhibit high survival generally contribute more to population dynamics than younger individuals [39] i.e. they are considered more ‘valuable’. A similar pattern is true also for our study species, in which older females have higher hatching success than younger ones [29], and they are also more attractive as partners in brood-rearing coalitions [30]. For example, Öst et al. [19] found that the long-term population growth rate of female eiders was most sensitive to relative changes in the survival of the prime reproductive-age individuals (≥ 5 years) regardless of population status (declining or stable). Therefore, it is not surprising that a slightly female-biased duckling sex ratio at hatching and sex-specific survival among younger individuals had a minor effect on population dynamics and, consequently, on ASR in the study populations. We note, though, that in the present study, the survival of adult females remains constant regardless of age and therefore, the results may not be applicable to species in which female survival remarkably decreases with increasing age. For the eider, constant survival is a reasonable assumption because most individuals in the study area die at their prime age [18] without reaching a theoretical expected lifespan of about 21 years [40]. It is also important to remember that the present study is based on an indirect estimate of ASR, which assumes that the population has reached its stable age distribution, and should therefore be considered the hypothesized ASR [41].

### Potential mechanisms for shifts in adult sex ratio

Overall, our findings indicate that the observed change to a male-biased sex ratio in adult eiders in the northern Baltic Sea [15] can indeed be due to a lower (2–32 percentage points) survival of females than males among experienced breeders in the population. The most likely reason for the reduced female survival is increased predation pressure on incubating females that stay on the nests during incubation, thus being more exposed to predation than males. Increased predation pressure particularly by a native avian predator (the white-tailed sea eagle) has paralleled the decline of eiders during the same time period in the Tvärminne population [15,18,42,43]. Moreover, introduced mammalian predators, such as the American mink (*Neovison vison*) and the raccoon dog (*Nyctereutes procyonoides*), prey on incubating females [18]. An alternative explanation for the reduced female survival is reproductive costs due to egg production and incubation. For birds, female survival decreases with increasing reproductive output [13], indicating that reproduction is costly. For example, Alisauskas and Devink [44] reported that there tends to be a trade-off between egg mass and clutch size in sea ducks, including eiders, which greatly rely on endogeneous nutrients during incubation. While the survival of female eiders decreases with decreasing body condition [18], the mean body condition of females at hatching has actually increased during the past decade in the study area (Öst et al., unpubl.), indicating that breeders are generally in good body condition. This suggests that reproductive costs may not have changed as dramatically as ASR during the past years and therefore, they may not be the main reason for the low female survival.

In addition to survival differences between sexes among experienced breeders, there are at least three other mechanisms that might lead to male-biased ASR. First, increasing predation pressure on ducklings or diseases during the brood-rearing period may disproportionately affect female offspring, as duckling mortality during their first month of life is female-biased [16]. To explore this possibility further, we conducted additional population simulations with the survival of male ducklings set to be arbitrarily higher than that of female ducklings (*S*_j_ = 0.60 or 0.70 for males and 0.5 for females). Depending on the population fraction in question, these simulations increased the proportion of males among adult birds up to 51.4%–55.6% during the simulation period of 50 years (results not shown), suggesting that sex-specific differential survival during the first year of life is not likely to explain the observed male-biased ASR of eiders. Second, in addition to direct predation, breeding females may experience more stress due to increased male abundance and attendant harassment, which may reduce female breeding propensity and hatching success. Considerable male aggression has been reported in eiders, in which unmated males may aggregate into larger ‘harassment groups’ [45]. However, we could not point out that such aggression would affect the decision whether or not to breed in our study population (Öst et al., unpubl.) and it is therefore unlikely that male interference would affect the survival of adult females. Third, male-biased ASR in small and declining populations could arise through local-scale sex differences in dispersal if females recruit disproportionately into larger populations and are thereby lost from the breeding population [3,10]. One could argue that such patterns of dispersal may be enhanced by increasing predation pressure, as females might then opt to settle in high-density populations to dilute their predation risk. Furthermore, female fitness (including survival) may be reduced more than that of males by longer dispersal distances [46], a potential consequence of increased fragmentation of attractive high-density breeding sites as the population decline progresses. However, because eiders, just like most waterfowl, show male-biased dispersal [47,48], this mechanism is unlikely to explain the change in ASR for this species. Alternatively, male-biased dispersal (i.e. an influx of males from other populations) could also potentially explain the observed male bias in ASR. This mechanisms, in turn, is probably not important for eiders, which show increasing male dominance across a large spatial scale [15]. For example, the increasing male bias is also evident in the Danish wing survey data, i.e., the shift in ASR has occurred over the scale of the entire Baltic Sea/Wadden Sea flyway population [15]. Therefore, we see no reason for why males would prefer to immigrate into an already strongly male-biased population with poor average reproductive success. In general, sex-biased dispersal is a redundant explanation in our case, as we can parsimoniously explain the observed change in ASR with sex-specific adult survival.

Our study adds to the growing evidence that poor conservation status of threatened bird species is often due to an increase in female mortality (reviewed in [9]), be it by introduced species, rapidly recovering native apex predators or increased reproductive costs. Our results have important conservation implications because the ASR directly impacts population growth rate through the number of reproducing females. Failing to incorporate sex- and age-specific vital rates could lead to erroneous conclusions about which sex and age-class(es) to prioritise in conservation efforts, and may seriously overestimate population viability [12]. The importance of considering both sexes in population models when their demographic rates differ has recently been emphasised [28,38] and the present study echoes this view. As the eider has recently been classified as endangered in Europe due to its steep decline [49], we strongly urge tailored and coordinated conservation strategies to improve the survival prospects of breeding female eiders over the scale of the entire Baltic Sea.

## Supporting information

S1 Appendix R code used to simulate change in the adult sex ratio of eiders. (DOC)
