# Supplementary material for: Increased male bias in eider ducks can be explained by sex-specific survival of prime-age breeders
Source: PLoS One. 2018 Apr 10;13(4):e0195415. doi: 10.1371/journal.pone.0195415 (PMC5892891; doi:10.1371/journal.pone.0195415)
Supplement: S1 Appendix — (DOC) [file pone.0195415.s001.doc]

**Supporting Information**

**S1 Appendix.** R-code used to simulate change in adult sex ratio and to calculate stochastic population growth rate for the eider. The example uses the parameter estimates for Söderskär.

#########################################################################################

# R-CODE FOR:

# 1. Simulating vital rates based on their estimated means, estimation uncertainty,

# and annual variation

#

# 2. Examining changes in adult sex ratio and calculating stochastic population growth rate

# based on the simulated vital rates

#

# By: Satu Ramula and Andreas Lindén

#########################################################################################

# Load required packages

library(popbio)

library(MASS)

runs <- 100 # No. runs

tmax <- 50 # No. years

numvr <- 17 # No. vital rates

nrows <- 10 # No. rows in a matrix

##### Create variables to collect results

growth <- as.vector(c(rep(1,tmax))) # Annual growth from 1:tmax

mratio <- matrix(nrow = 1, ncol = tmax) # The proportion of males from 1:tmax

lambda.res <- matrix(nrow = runs, ncol = 1); # Mean lambda per run

mratio.res <- matrix(nrow = runs, ncol = tmax) # The proportion of males per run

###########################################################################

# Simulate vital rates (Söderskär)

###########################################################################

# Mean vector for ln(no. offspring) and logit(survival)

m <- c(-2.5111, 1.8131)

# Covariance matrices describing sampling variation (Sigma.m) and annual

# variation (Sigma.a) in vital rates

Sigma.m <- matrix(c(0.0959, 0.0036, 0.0036, 0.0078),2,2)

Sigma.a <- matrix(c(1.4694, 0.0684, 0.0684, 0.0032),2,2)

# The outer for-loop simulates average vital rates for each run

for (k in 1:runs){

# An initial population vector and total population size

n0 <- matrix(c(21,20,20,20,920,19,20,20,20,920), nrow = 10, ncol = 1, byrow = TRUE)

n <- n0/sum(n0)

# Averages for run k are drawn from a multivariate normal & normal distributions

m.k <- mvrnorm(n = 1, mu = m, Sigma.m, empirical = FALSE)

males.k<-rnorm(n=1, -0.11208, 0.05336^2) #Proportion of males among hatched ducklings

# The inner for-loop simulates time specific annual vital rates

for (i in 1:tmax){

# Fixed parameters

Su <- 0.50 # Juvenile survival

P2 <- 0.09 # Probability to reproduce as 2-year old

P3 <- 0.36 # Probability to reproduce as 3-year old

P4 <- 0.38 # Probability to reproduce as 4-year old

P5 <- 0.17 # Probability to reproduce as 5-year old

males.i<-rnorm(n=1, males.k, 0.06498^2) #annual variation in prop. of males

# Simulate vital rates (fecundity and survival) for current year

vital.i <- mvrnorm(n = 1, mu = m.k, Sigma.a, empirical = FALSE)

# Back-transformed no. offspring (F), proportion of males at hatching (R), and

# survival for females (S1-S5) and for males (SM1-SM5)

F <- exp(vital.i[1])

S1 <- exp(vital.i[2])/(1+exp(vital.i[2]))

S2 <- S1

S3 <- S1

S4 <- S1

S5 <- S1

R <- (exp(males.i))/(1+exp(males.i))

SM1<-S1

SM2<-S1

SM3<-S1

SM4<-S1

SM5<-0.92

# Form the transition matrix (A)

ratet<- c(S1=S1, P2=P2, F=F, R=R, Su=Su, S2=S2, P3=P3, S3=S3, P4=P4, S4=S4,

P5=P5,S5=S5, SM1=SM1, SM2=SM2, SM3=SM3, SM4=SM4, SM5=SM5)

rate.vr<- list(S1=S1, P2=P2, F=F, R=R, Su=Su, S2=S2, P3=P3, S3=S3, P4=P4, S4=S4,

P5=P5, S5=S5, SM1=SM1, SM2=SM2, SM3=SM3, SM4=SM4, SM5=SM5)

mat.el <- expression(

0, S1, 0, 0, 0, 0, 0, 0, 0, 0,

P2*F*(1-R)*Su, 0, S2, 0, 0, P2*F*R*Su, 0, 0, 0, 0,

(P2+P3)*F*(1-R)*Su, 0, 0, S3, 0, (P2+P3)*F*R*Su,0, 0, 0, 0,

(P2+P3+P4)*F*(1-R)*Su, 0, 0, S4, 0, (P2+P3+P4)*F*R*Su, 0, 0, 0, 0,

(P2+P3+P4+P5)*F*(1-R)*Su, 0, 0, 0, S5, (P2+P3+P4+P5)*F*R*Su, 0, 0, 0, 0,

0, 0, 0, 0, 0, SM1, 0, 0, 0, 0,

0, 0, 0, 0, 0, 0, SM2, 0, 0, 0,

0, 0, 0, 0, 0, 0, 0, SM3, 0, 0,

0, 0, 0, 0, 0, 0, 0, 0, SM4, 0,

0, 0, 0, 0, 0, 0, 0, 0, 0, SM5)

A <- matrix(sapply(mat.el, eval, rate.vr), nrow = 10, byrow = FALSE)

################################################################################

# Calculate stochastic population growth rate

################################################################################

n <- A %*% n

growth1 <- sum(n)

growth[i] <- sum(n)

n <- n/growth1

mratio[,i] <- sum(n[6:10])/sum(n) # Collect prop. males for each time step

} # Close inner loop (for i in 1:tmax)

lambda.res[k,]<- mean(log(growth)) # Mean lambdas from different runs

mratio.res[k,] <- as.vector(mratio) # Proportion of males from different runs

} # Close outer loop (for k in 1:runs)

########################################################

# OUTPUT: Estimated ln-growth rate (lambda), its standard error (SE),

# lower and upper 95% confidence limits (CL.lo, CL.hi)

########################################################

lambda <- mean(lambda.res[,1])

SE <- sqrt(var(lambda.res[,1]))

OUTPUT <- list(lambda = lambda, SE = SE, CL.lo = lambda-1.96*SE, CL.hi= lambda+1.96*SE)

print(OUTPUT)

print(colMeans(mratio.res)) # Mean proportion of males across different runs

SE.mratio <- sqrt(var(mratio.res))
